# The ETRAMP Family Member SEP2 Is Expressed throughout *Plasmodium berghei* Life Cycle and Is Released during Sporozoite Gliding Motility

**DOI:** 10.1371/journal.pone.0067238

**Published:** 2013-06-28

**Authors:** Chiara Currà, Marco Di Luca, Leonardo Picci, Carina de Sousa Silva Gomes dos Santos, Inga Siden-Kiamos, Tomasino Pace, Marta Ponzi

**Affiliations:** 1 Istituto Superiore di Sanità, Dipartimento di Malattie Infettive, Parassitarie ed Immunomediate, Roma, Italy; 2 Instituto de Medicina Molecular, Lisboa, Portugal; 3 Institute of Molecular Biology and Biotechnology, FORTH, Heraklion, Greece; University of Bern, Switzerland

## Abstract

The early transcribed membrane proteins ETRAMPs belong to a family of small, transmembrane molecules unique to *Plasmodium* parasite, which share a signal peptide followed by a short lysine-rich stretch, a transmembrane domain and a variable, highly charged C-terminal region. ETRAMPs are usually expressed in a stage-specific manner. In the blood stages they localize to the parasitophorous vacuole membrane and, in described cases, to vesicle-like structures exported to the host erythrocyte cytosol. Two family members of the rodent parasite *Plasmodium berghei*, *uis3* and *uis4*, localize to secretory organelles of sporozoites and to the parasitophorous membrane vacuole of the liver stages. By the use of specific antibodies and the generation of transgenic lines, we showed that the *P. berghei* ETRAMP family member SEP2 is abundantly expressed in gametocytes as well as in mosquito and liver stages. In intracellular parasite stages, SEP2 is routed to the parasitophorous vacuole membrane while, in invasive ookinete and sporozoite stages, it localizes to the parasite surface. To date SEP2 is the only ETRAMP protein detected throughout the parasite life cycle. Furthermore, SEP2 is also released during gliding motility of salivary gland sporozoites. A limited number of proteins are known to be involved in this key function and the best characterized, the CSP and TRAP, are both promising transmission-blocking candidates. Our results suggest that ETRAMP members may be viewed as new potential candidates for malaria control.

## Introduction

Malaria is one of the oldest and most frequently occurring infectious diseases in humans. It is caused by *Plasmodium* parasite, an obligate intracellular protozoa transmitted through the bite of an infected female mosquito. Each year malaria disables hundreds of millions of people and kills more than half a million people worldwide. The rapid emergence and spread of drug resistant parasites in the endemic areas makes the development of new drugs/vaccines against this disease a health priority. *Plasmodium* undergoes a complex multi-stage life cycle with two hosts, the vertebrate and the mosquito vector of the genus *Anopheles*. In the vertebrate host the parasite either multiplies asexually or differentiates to sexual stages, the male and female gametocytes. These gamete precursors develop in the blood, but gametogenesis and gamete fertilization only takes place after the uptake of a blood meal by the mosquito. The resulting zygote develops into a motile ookinete, which invades the midgut epithelium and next forms an oocyst. Repeated mitotic divisions in the oocyst finally lead to the formation of thousands of sporozoites. These have the ability to recognize and invade different cell-types. After the exit of the sporozoites from the oocyst [1], [2] they invade the mosquito salivary glands. Sporozoites are then injected into a mammalian host during a mosquito blood meal and are rapidly transported to the liver, where they invade the hepatocytes. Here, they multiply inside a parasitophorous vacuole (PV), resulting in the release of thousands of merozoites. These invade erythrocytes and thus the asexual multiplication in the blood is commenced.

Ultrastructural studies [3] identified a three-membrane pellicle at the surface of sporozoites, the outer plasma membrane, derived from the oocyst plasma membrane and a double inner membrane, the so-called inner membrane complex (IMC). A limited number of proteins have been detected so far on the sporozoite surface. One of the best characterized is the multifunctional circumsporozoite protein (CSP) [4], [5], [6]. It is involved in gliding motility [7], a form of substrate-dependent cell locomotion characteristic of Apicomplexa, as well as in mosquito salivary gland binding [8], [9] and in the inhibition of protein synthesis of the infected liver cell [10], [11]. A second, well-studied surface protein implicated in sporozoite motility and in liver cell invasion is the thrombospondin-related anonymous protein (TRAP) [12]. TRAP contains a thrombospondin type I repeat (TRS), which mediates interactions with the substrate. This motif is shared with the thrombospondin-related sporozoite protein (TRSP) [13] also involved in liver cell invasion [14]. Both CSP and TRAP are in part routed to secretory organelles (micronemes) and are secreted during motility and invasion [15].

Moreover, two members of the conserved early transcribed membrane protein (*etramp*) gene family, referred to as *uis3* [13], [16] and *uis4* [17] are upregulated in salivary gland sporozoites of the rodent parasite *P. berghei*. Their gene products localize to the micronemes of salivary gland sporozoites and to the PVM in infected hepatocytes and are essential for early liver stage development [17], [18].

ETRAMPs are small molecules that share a signal peptide followed by a short lysine-rich stretch, a transmembrane domain and a variable, highly charged C-terminal region. ETRAMPs, described so far, are expressed/upregulated in a stage-specific fashion. They were first characterized in blood stages of the human parasite *P. falciparum* [19] and the rodent model *P. berghei* [20] where they mainly localize to the PVM. Members of this family were also detected in vesicle-like structures beyond the PVM [21], [22]. ETRAMPs form large protein arrays both in *P. falciparum* [23] and *P. berghei* [21]. Complex formation requires a membrane milieu [23] and the presence of the charged C-terminal region [21].

In *P. berghei* three *etramp* family members, referred to as *sep1-3* (PBANKA_052480, PBANKA_052420 and PBANKA_050110, respectively), localize to the subtelomeric portions of chromosome 5 [24]. They share the upstream regulatory region and part of the coding region including the transmembrane domain, while they differ in the C-terminal charged region and the 3′UTR. In asexual blood stages SEP2 and SEP3 localize to the PVM and to vesicle-like structures exported to the erythrocyte cytosol [21], while SEP1 is mainly confined to the PVM [20].

In a previous study [25], a parasite mutant was characterized, harboring a terminal deletion of chromosome 5, which includes *sep1* but not *sep2* and *sep3*. This parasite line, obtained through repeated cycles of asexual multiplication in mice in the absence of mosquito transmission, is not affected in blood stage development, indicating that the subtelomeric gene products, including SEP1, are dispensable. This was also confirmed by the generation of a transgenic line harboring a 50-kb terminal deletion of chromosome 5, which included the endogenous *sep1* [26]. Repeated attempts to disrupt *sep2* and *sep3* were instead unsuccessful [21], suggesting an essential role of their gene products.

In this study we investigated the expression of SEP2 and SEP3 in the mosquito vector using transgenic lines and specific antibodies. We showed that SEP2 is highly expressed throughout the mosquito cycle, while SEP3 is a low-abundance protein. At the sporozoite stage SEP2 localizes to the cell surface and is in part released during gliding motility of salivary gland sporozoites. Upon hepatocyte infection, SEP2 is readily detected at the periphery of the exoerythrocytic forms, suggesting an additional role in liver stages.

## Results

### SEP2 and SEP3 are Expressed in Blood Stages and Ookinetes

**Figure 1:**
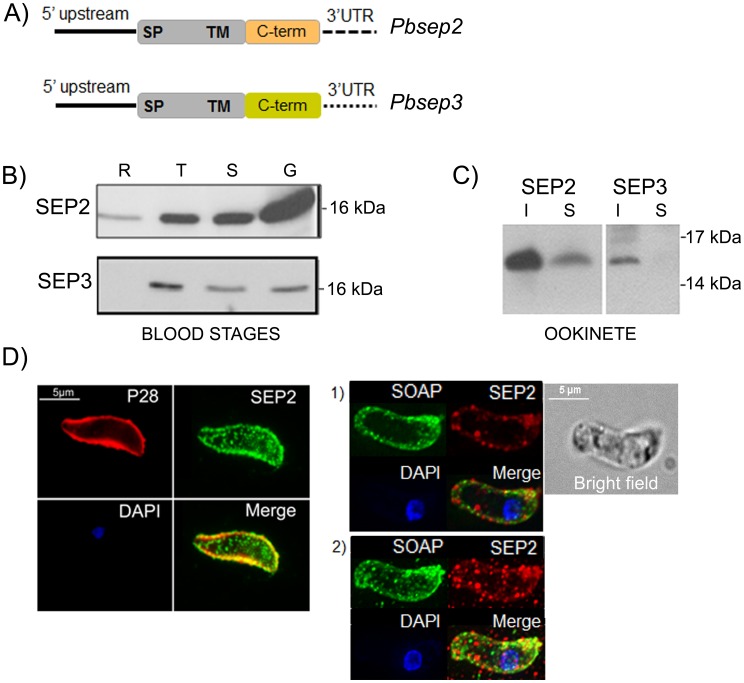
SEP2 and SEP-3 are integral membrane proteins expressed in blood stages and ookinetes. A) Schematic representation (not drawn to scale) of *Pbsep2* and *Pbsep3* loci. B) *P. berghei* rings (R), trophozoites (T), schizonts (S) and gametocytes (G) were analyzed by western blot using αSEP2 and αSEP3 immune sera. The amount of protein loaded in each lane was assessed by Bradford. C) soluble (S) and insoluble (I) fractions prepared from purified ookinetes were analyzed by western blot using αSEP2 and αSEP3 immune sera. Both proteins are mainly detected in the insoluble fraction. D) Specific antibodies detect SEP2 in dot-like structures inside the ookinete but also close to the parasite periphery, as shown by partial co-localization with the ookinete surface protein P28 (left panel). SEP2 does not co-localize with the micronemal protein SOAP (right panel). A single section (1) and a stack of the same ookinete (2) are shown. The sample was imaged in a DeltaVision Elite deconvolution microscope and one single section through the middle of the ookinete is shown. DNA was labeled with DAPI (blue).

We analyzed the expression of *sep*2 and *sep*3 at different time points of a synchronous *P. berghei* infection, using specific mouse immune sera [21] raised against the variable C-terminal portions (Fig. 1A). Western blot analysis was performed on parasite extracts obtained from rings at 6 hours post invasion (hpi), trophozoites (13 hpi) and gametocytes (28 hpi). Mature schizonts, containing the erythrocyte invasive forms (merozoites), were collected from cultured parasites, since this stage is sequestered in *in vivo* infections. SEP proteins were detected both in asexual and sexual stages. Interestingly, SEP2 exhibited a remarkable increase in its relative abundance in sexual stages (Fig. 1B)

The presence of SEP proteins in gametocytes led us to investigate their expression in fractionated protein extracts from purified ookinetes. As shown in Figure 1C, protein bands of the expected size of around 16 kDa were mainly detected in the insoluble fractions, indicating that SEP2 and SEP3 localize to membranous compartments of the ookinete, as it is also the case of the asexual blood stages.

Subcellular localization was determined only for SEP2, since antibodies raised against SEP3 do not recognise the protein in the native form [21]. In IFA on fixed ookinetes SEP2 was detected in punctate structures inside the cytoplasm and close to the ookinete surface. This distinct localization was also confirmed by double labelling with antibodies against the P28 antigen, which marks the ookinete surface (Fig. 1D, left panel). The punctate labelling pattern may suggest that SEP2 localizes to the micronemes, secretory organelles likely involved in host-cell recognition, binding, and motility. We therefore performed double labeling with an antibody directed against the micronemal protein SOAP [27], followed by deconvolution microscopy (Fig. 1D, right panel). Representative images reveal that the two proteins localize to distinct dots, suggesting that ookinetes contain vesicle-like structures, which differ in protein composition.

### Expression Profile of Chimeric SEP2 and SEP3 in Mosquito Stages

**Figure 2:**
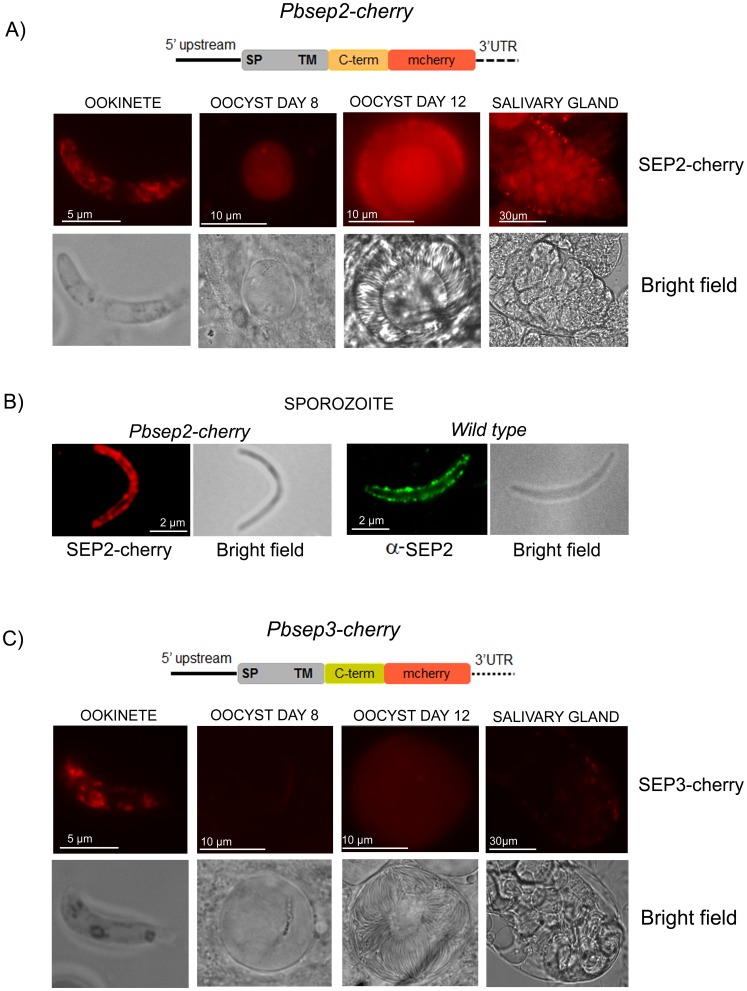
Mosquito stages of *P. berghei* transgenic lines harboring *sep2* or *sep3* fused to mCherry. Transgenic lines, *Pbsep2-cherry* and *Pbsep3-cherry*, harboring an integrated copy of *sep2* or *sep3* fused with the fluorescent reporter mcherry (drawn in figure), were used to infect mosquitoes. A) SEP2 is detected in all mosquito stages with a peak of expression in mature oocysts (day 12 after infection). The protein fusion (SEP2-cherry) is detected both in the sporoblast and on the sporozoites being formed. B) Higher magnification of *Pbsep2-cherry* salivary gland sporozoite reveals that SEP2-cherry localizes to the parasite periphery. IFA with α-SEP2 immune serum on non-permeabilized wild type sporozoites shows a similar fluorescence pattern. C) In young oocysts of the *Pbsep3-cherry* line, SEP3-cherry localizes only to a pigmented area. In mature oocysts, it shows a faint diffuse signal. Salivary gland sporozoites are weakly fluorescent.

In order to follow the expression of SEP2 and SEP3 in mosquito stages, we generated genetically modified *P. berghei* lines (*Pbsep2-cherry* and *Pbsep3-cherry*), which contain an integrated full-length extra-copy of *sep2* or *sep3* fused to the mCherry fluorescent reporter (SEP2-cherry and SEP3-cherry). Inspection of live *Pbsep2-cherry* parasites by fluorescence microscopy revealed a strong mCherry-based fluorescence in ookinetes, sporulating oocysts, and salivary gland sporozoites (Fig. 2A). In mature sporozoites, the majority of the mCherry-based fluorescence was found close to the parasite periphery (Fig. 2B, left panel). A similar flurescence pattern was observed using SEP2-specific antibodies on non-permeabilized salivary gland sporozoites (Fig. 2B, right panel), suggesting that the C-terminal portion of SEP2 is exposed to the cell surface.

As shown in Fig. 2C, *Pbsep3-cherry* ookinetes displayed a pattern of fluorescence similar to that observed in *Pbsep2-cherry* line. Conversely, young oocysts (8 days) displayed only a very faint signal confined to a small pigmented area, most probably corresponding to a residual ookinete-derived compartment. In mature oocysts (12 days) and in salivary gland sporozoites we detected only a diffuse very weak fluorescence.

### Downstream Regulatory Regions are Involved in the Expression Level of sep2 and sep3 in Mosquito Stages

**Figure 3:**
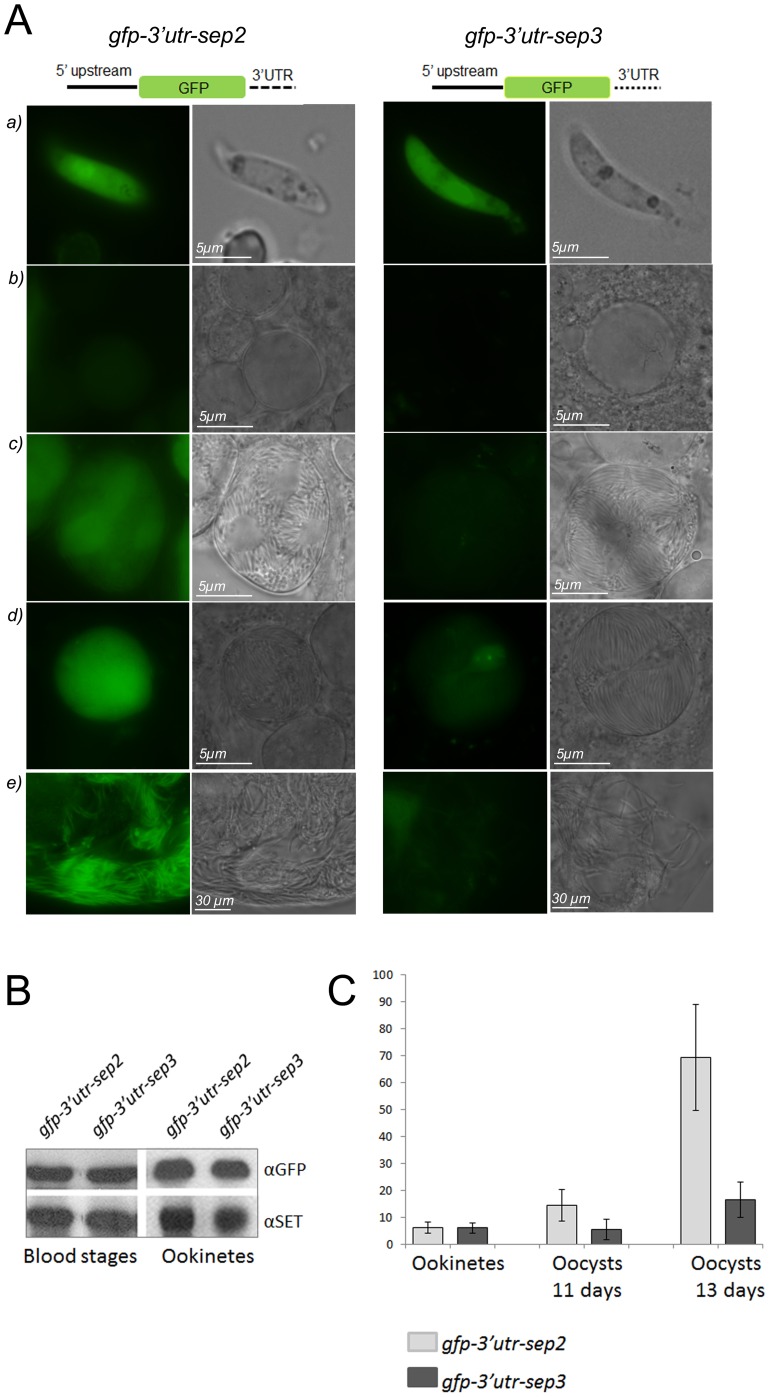
3′UTRs of *sep2* and *sep3* modulate the expression level of GFP. A) Transgenic lines expressing the GFP reporter under the control of the upstream regulatory region common to *sep* genes and the 3′UTR specific for either *sep2* (*gfp-3′utr-sep2*, left panel) or *sep3* (*gfp-3′utr-sep3*, right panel) were analyzed *in vivo* in ookinetes (a), young oocysts, 6 days upon infection (b), mature oocysts, 11 and 13 days upon infection (c and d respectively) and salivary gland sporozoites (e). The 3′UTR of *sep2* is sufficient to sustain the expression of GFP in all mosquito stages while the 3′UTR of *sep3* leads to a GFP fluorescence evident in ookinetes and very weak in oocysts and sporozoites. A schematic representation of the constructs is drawn in figure, not to scale. B) GFP level in mixed blood stages and ookinetes of the *gfp-3′utr-sep2* and *gfp-3′utr-sep3* lines was analyzed by western blot. Protein extracts were normalized using antibodies against the nuclear protein SET, expressed throughout parasite life cycle. C) Normalized GFP fluorescence intensity (y-axis) of the two transgenic lines (10 cells per each stage), is plotted.

*Sep* genes share the upstream regulatory region, while they differ in their 3′ UTRs [20], [21]. In order to establish whether the downstream regulatory regions have a role in the control of the expression level of these genes, we infected *Anopheles stephensi* mosquitoes with two transgenic lines, *gfp-3′utr-sep2* and *gfp-3′utr-sep3* [21], containing a stably integrated *gfp* reporter under the control of the 1.2-kb upstream regulatory region common to *sep* genes and the 3′UTR specific for either *sep2* or *sep3*. In asexual blood stages these transgenic lines express GFP at a comparable level, indicating that the specific 3′UTRs have no significant regulatory role [21]. In this study, GFP expression of both *gfp-3′utr-sep2* and *gfp-3′utr-*sep3 lines was visually inspected during the mosquito cycle (Fig. 3A). Infected mice were first used to feed mosquitoes and then bled to produce ookinetes *in vitro*. As shown in Fig. 3A, fully developed ookinetes of the two transgenic lines display a comparable intensity of GFP-specific fluorescence. This was also confirmed by western blot analysis of protein extracts from purified ookinetes and mixed blood stages probed with GFP-specific antibodies (Fig. 3B).

In the *gfp-3′utr-sep2* line (Fig. 3A, left panel), GFP fluorescence was clearly detected during oocyst maturation (8, 11, 13 days after the blood meal) with an increased intensity in mature oocysts (13 days). A strong GFP-specific signal was also detected in salivary gland sporozoites (22 days). When the GFP was expressed under the control of the 3′ UTR specific for *sep3* (*gfp-3′utr-*sep3 line), only mature oocysts and salivary gland sporozoites displayed a faint fluorescence (Fig. 3A, right panel).

Quantitative analysis of fluorescence intensity (Fig. 3 C) indicated that the GFP signal of mature oocysts of the *gfp-3′utr-sep2* line was at least 4 times higher than that of oocysts of the *gfp-3′utr-sep3* line. These results are consistent with the expression profile of the SEP-cherry fusions.

### SEP2 is Released during Sporozoite Gliding Motility

**Figure 4:**
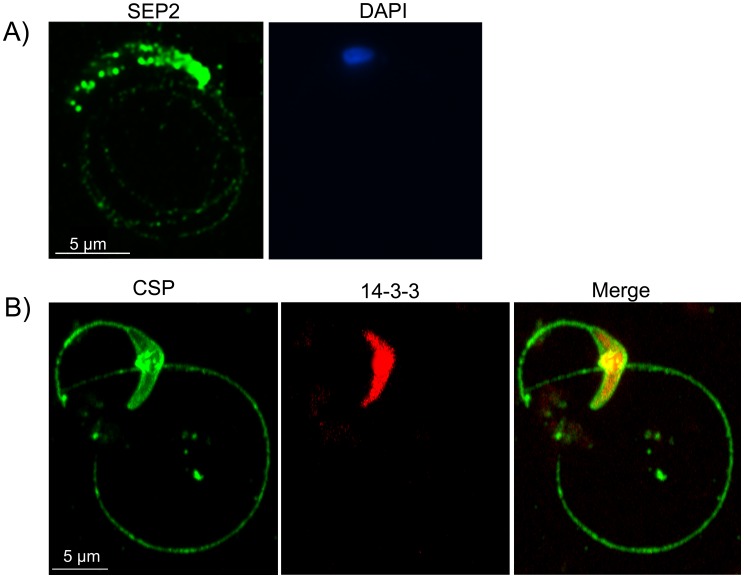
SEP2 is secreted during gliding motility of salivary gland sporozoites. A) Wild type salivary gland sporozoites were allowed to glide on glass microscopy slides coated with αSEP2 antibodies. Subsequent IFA with a secondary fluorescent antibody revealed that SEP2 is secreted in the trails left by the parasite during its motility. SEP2 is also detected on the surface of the sporozoite in dot-like structures. B) A similar gliding assay using the αCSP and α14-3-3 antibodies, shows that CSP is uniformly localized on the surface of the parasite and in the trails, while 14-3-3 localizes to the parasite cytoplasm.

SEP2-cherry fluorescence in live sporozoites as well as IFA with specific antibodies (Fig. 2B) suggested that SEP2 is routed to the parasite surface. We then decided to investigate the localization of SEP2 during sporozoite gliding motility *in vitro*. Sporozoites were collected from salivary glands and incubated on glass slides treated as described in the Materials and Methods section. αSEP2 antibodies layered on glass slides are expected to bind SEP2 if the protein is released during gliding motility. In a parallel assay, we layered mouse antibodies against the sporozoite surface protein CSP (positive control) and a rabbit polyclonal immune serum against the cytoplasmic protein 14-3-3 (negative control) on the same glass slide. IFA was then performed on fixed samples using either αSEP2 or αCSP/14-3-3 antibodies to outline trails of released proteins. As shown in Fig. 4, SEP2 was detected at the periphery of sporozoite and in characteristic circles behind the parasite, indicating that this protein is released during gliding motility. SEP2 displayed a surface, patchy distribution, suggesting that it partitions into well-defined membrane subdomains. At difference, CSP protein uniformly covered the periphery of salivary gland sporozoites and the trails left behind by gliding parasites. 14-3-3-specific signal remained confined to the sporozoite cytosol.

### SEP2 is Expressed in the Liver Stages

**Figure 5:**
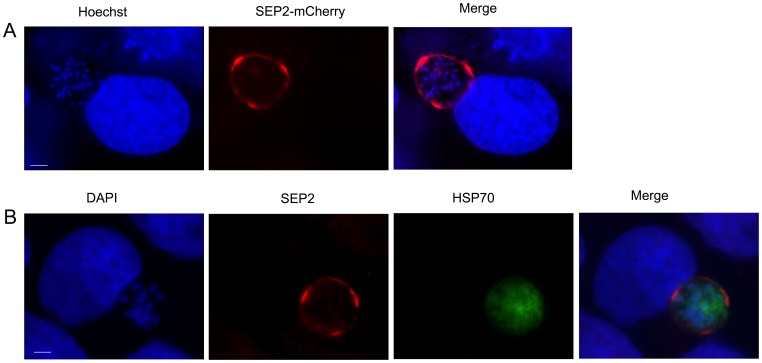
SEP2 is expressed in liver stages and localizes to the parasite periphery. **A)** The chimeric protein SEP2-cherry localizes to the periphery of *P. berghei* liver stages at 48 hpi. B) A similar fluorescence pattern is also observed in IFA using αSEP2 antibodies on wild-type sporozoites. Parasites were co-stained with antibodies raised against the parasite 75-kDa heat-shock protein (HSP70). Bars correspond to 5 µm.

We next asked whether SEP2 is also expressed in liver stages. To answer this question, we infected the Huh7 hepatocyte cell line with salivary gland sporozoites of the line *Pbsep2-cherry* and monitored the infection at 48 hpi. As shown in Figure 5A, the chimeric protein SEP2*-*cherry decorated the periphery of the parasite. We obtained a similar fluorescence pattern using SEP2-specific antibodies on the wild type *P. berghei* parasites (Fig. 5B). Parasites were co-stained with antibodies raised against the parasite 75-kDa heat-shock protein (HSP70).

## Discussion

ETRAMP family members are small integral membrane proteins specific to *Plasmodium* genus. They share conserved structural features, i.e. the presence of a N-terminal signal peptide a short lysine-rich region followed by a transmembrane domain and a charged C-terminal region. They are expressed in a stage-specific manner during parasite life cycle. Some of them such as ETRAMPS 2, 10.1, 11.1, 11.2, 12, and 14 were detected at the PVM of *P. falciparum* blood stages [19], [23]. Two family members, UIS3 and UIS4, were previously identified as pre-erythrocytic stage-specific proteins in the rodent malaria parasites *P. yoelii* and *P. berghei* [16], [28], [29]. Both UIS3 and UIS4 are upregulated at the sporozoite stage and localize to the PVM of the liver stages, and each one is essential for liver-stage development [17], [18], [30].

The transcription profile of nine *P. yoelii etramp* genes was recently investigated [31] throughout the parasite life-cycle. It was shown that the *etramps* of *P. yoelii* are transcribed in mixed blood stages except UIS3, UIS4 and a third gene, *PY03365*, which are expressed in sporozoites. We demonstrated previously [20], [21] that three *P. berghei* family members, SEP1-3, are expressed in all asexual blood stages. SEP1 is dispensable for parasite development [25], [26] and localizes to the PVM [20]. SEP2 and SEP3 are likely essential proteins and localize to the PVM and to exported vesicular structures [21]. In this study we showed that the expression level of SEP3 in gametocytes is similar to that observed in asexual parasites, while SEP2 is more abundant in sexual stages.

At the ookinete stage, specific antibodies detected SEP2 in dot-like structures mainly at the periphery of the cell. These structures do not coincide with the micronemes containing the protein SOAP [27], [32], suggesting the presence of distinct vesicle-like structures, which differ in protein composition. We could not determine the subcellular localization of SEP3, since the specific antibodies failed to recognize the protein in IFA.

We further characterized the expression of SEP2 and SEP3 in mosquito stages, following SEP-mCherry fluorescence in *in vivo* infections of transgenic parasite lines harboring the chimeric genes. We detected an intense SEP2-specific fluorescence in oocysts and in salivary gland sporozoites while SEP3-specific fluorescence was much less intense in all mosquito stages.

This difference in protein abundance was unexpected since the *P. berghei sep* genes share the upstream promoter region. We then hypothesized that the different expression profile of *sep2* and *sep3* in the mosquito stages may be due to a regulatory role of the specific 3′UTRs. To verify this we analyzed two transgenic lines expressing the GFP under the control of the common *sep* promoter and the 3′UTR specific for either *sep2* or *sep3* [21]. This resulted in a different abundance of the GFP reporter, similar to that of the cognate proteins expressed as mCherry fusions. Our previous studies indicated that the specific 3′UTRs do not play any regulatory role in asexual blood stages [21] while they are probably determinants of protein abundance in mosquito stages.

Using two different experimental approaches, either imaging parasites expressing SEP2-cherry-fusion or performing IFA with specific antibodies, we showed that SEP2 is routed to the surface of the salivary gland sporozoites. Furthermore, SEP2 is secreted during gliding motility of these invasive forms and localizes to the periphery, most likely the PVM, of liver stages. Overall these data suggest that SEP2 may play a role in the infection of liver cells.

To date, few proteins are known to be secreted during sporozoite gliding and the most studied are the CSP and TRAP. The sporozoite invasion-associated protein (SIAP), has been recently described [33] as specifically involved in gliding. In fact, deletion of *siap* dramatically affects sporozoite mobility. Unfortunately, the inability to delete the *sep2* gene using standard methodologies, did not allow us to study directly the function of SEP2 in sporozoite gliding and invasion, although novel conditional mutagenesis methods [34] may make this possible in the future.

In conclusion, we show that SEP2, a member of the ETRAMP family of *P. berghei*, previously detected in asexual blood stages [21], is also expressed in gametocytes, ookinetes, oocysts, salivary gland sporozoites and liver stages. This is in contrast to the stage-specific expression of the ETRAMPs characterized so far. Moreover, the subcellular localization of SEP2 depends on the parasite developmental stage. In intracellular blood and likely liver stages, the protein localizes to the PVM while in the sporozoite invasive form it is routed to the parasite surface. Sporozoite proteins [35] have been recognized as attractive targets for antimalarial vaccine development [36], [37]. To date, CSP is the only *Plasmodium* protein shown to confer protection to immunized individuals and the protein has been included in formulations for vaccines, of which the most advanced is the RTS’S vaccine [38]. In this context, SEP2 warrants further studies since our data suggest that it may be involved in key processes and localizes to the surface of liver invasive forms. Even though the identification of a *P. falciparum* ortholog is made difficult by the poor conservation of the protein sequence between family members, the analysis of the ETRAMPs expression profiles may guide this search.

## Materials and Methods

### Ethics Statement

The animal work has been approved by the Service for Biotechnology and Animal Welfare of the Istituto Superiore di Sanità (National Institute of Health) and has been authorised by the Italian Ministry of Health, according to the Legislative Decree 116/92, which implemented in Italy the European Directive 86/609/EEC on laboratory animal protection. Animals used in this study were housed and treated according to Legislative Decree 116/92 guidelines and animal welfare has been routinely checked by veterinarians from the Service for Biotechnology and Animal welfare.

### Parasites Maintenance and Purification

The gametocyte producer *P.berghei* ANKA clone 8417HP was maintained in Swiss mice. Synchronous infections and purification of parasite blood stages were performed as described [39]. *In vitro* cultures of ookinetes were performed as described [40].

### Western Blot Analysis

Western Blot analysis was performed using MINI TRANS-BLOT® Bio-Rad apparatus at constant voltage (100 V) for 30 min, in transfer buffer (20% methanol, Tris 0.025 M, Glycine 0.192 M) onto Protran 0.22 microns membrane (Whatman). Primary and HRP-conjugated secondary antibodies were incubated one hour in PBS-Tween (0.05%) at room temperature. The membrane was developed using the ECL system (SuperSignalWest Pico, Thermo Scientific) according to manufacturer’s instructions.

### Immune Sera

Working dilutions of antibodies used in this study were: mouse polyclonal αSEP2 and αSEP3 immune sera, 1∶100 in IFA, 1∶1000 in western blot; mouse monoclonal αCSP (supplied by Maria Mota) 1∶400 in IFA; mouse monoclonal αGFP (Roche), 1∶1000 in western blot; mouse polyclonal αHSP70 [41], 1∶300 in IFA; rabbit polyclonal α14-3-3 (supplied by Marco Lalle) 1∶200 in IFA; rabbit polyclonal αSET [42], 1∶5000 in western blot; mouse monoclonal αP28 (supplied by Robert Sinden) 1∶400 in IFA; mouse polyclonal αSOAP antiserum (supplied by Robert Sinden) 1∶100 in IFA.

### Indirect Immunofluorescence Assay (IFA)

Purified ookinetes or salivary gland sporozoites were fixed on glass slides for 1 hour in 4% paraformaldehyde/0.0075% glutaraldehyde. After 3 washes in PBS, parasites were treated with 0.1% Triton X-100 in PBS and then blocked 1 hour at room temperature with 3% PBS/BSA. Parasites were incubated 1 hour with the primary antibody, washed several times in PBS and then incubated 1 hour with the fluorescein- or rodamin-conjugated goat αmouse or αrabbit secondary antibodies (1∶400 dilution). The cell nuclei were labeled with DAPI. The specificity of the immune sera was checked in parallel using pre-immune sera. The double-labeling with SEP2 and SOAP was performed on fresh purified ookinetes. All steps used PBS, pH 4.5. The ookinetes were fixed in 4% paraformaldehyde, 0.1% Triton X-100 in PBS, followed by incubation with the primary antibodies overnight at +4°C. Secondary antibodies were αmouse conjugated with Alexa Fluor 488 and αrabbit Alexa Fluor 568. Controls were included to exclude non-specific binding of the secondary antibodies; they were all negative. The samples were imaged in a Delta Vision Elite deconvolution microscope from Applied Precision. The images were deconvoluted using the softWoRx 5.5 built in module and further processed using Image J.

### Quantitative Image Analysis

To measure GFP fluorescence, averages of normalized intensity values of at least ten cells were calculated for each time point and plotted on a graph. For these experiments all parameters during image acquisition were the same. A region was drawn around each cell to be measured and around an area without fluorescent objects to be used for background subtraction. The net integrated intensity for each cell was measured using Image J.

### Mosquito Transmission Assay

A long-established laboratory mosquito strain of *Anopheles stephensi* was reared in a climatic chamber at 26±1°C, 70–80% relative humidity and a photoperiod of 12∶12 h light/dark. Three to five- day old female mosquitoes were used for feeding on infected mice with *P. berghei* ANKA and maintained in 35×35×35 cm steel frame cages, covered by a cotton net of 96 meshes/cm^2^ and provided *ad libitum* with a solution of 5% glucose both before and after blood meal. Twenty four hours before blood feeding, the glucose solution was replaced with distilled water to encourage engorgement.

### Gliding Motility Assay

Gliding assay was performed as described in [43]. To visualize trails left during sporozoite gliding motility, 3×10^4^ sporozoites (per well) were centrifuged for 5 min at 2000 rpm on a glass coverslip coated with αCSP or αSEP2 antibodies and incubated for 60 min at 37°C, in the presence of RPMI medium (Gibco/Invitrogen) supplemented with 10% FBS (Gibco). Coverslips were then fixed in 4% paraformaldehyde and stained with αCSP and αSEP2 antibodies, respectively, following standard IFA procedures. In control experiment glass coverslip was supplemented with both αCSP and α14-3-3 antibodies.

### Transfection Constructs

The procedure for constructing transfection plasmids, including plasmid maps and PCR primers, are described in Materials and Methods S1.

### Cultivation and Infection of Liver Cells

Cells of the Huh7 human hepatoma cell line [44] were cultured in RPMI medium supplemented with 10% fetal calf serum, 1% non-essential amino acids, 1% penicillin-streptomycin, 1% glutamine, and 10 mM HEPES (pH7) (all from Gibco/Invitrogen) and were maintained at 37°C under 5% CO2. For infection, *P. berghei* sporozoites obtained from freshly dissected mosquito salivary glands were added to cell monolayers seeded 24 hours earlier on coverslips in complete medium and used when confluence was ∼80–90%. Cell culture plates were centrifuged for 5 min at 1.800 *g* to promote contact of the parasites with the Huh7 cell monolayer.

## Supporting Information

Materials and Methods S1 ***Pbsep2-cherry*** **and** ***Pbsep3-cherry*** **transfection constructs.** The supporting file includes a detailed description of the cloning strategy used to construct *Pbsep2-cherry* and *Pbsep3-cherry* plasmids, plasmid maps and primers used for PCR amplifications.
(DOC)
