# Supplementary material for: The ETRAMP Family Member SEP2 Is Expressed throughout Plasmodium berghei Life Cycle and Is Released during Sporozoite Gliding Motility
Source: PLoS One. 2013 Jun 28;8(6):e67238. doi: 10.1371/journal.pone.0067238 (PMC3696012; doi:10.1371/journal.pone.0067238)
Supplement: Materials and Methods S1 — Pbsep2-cherry and Pbsep3-cherry transfection constructs. The supporting file includes a detailed description of the cloning strategy used to construct Pbsep2-cherry and Pbsep3-cherry plasmids, plasmid maps and primers used for PCR amplifications. (DOC) [file pone.0067238.s001.doc]

**Materials and Methods S1**

***Pbsep2-cherry* and *Pbsep3–cherry* transfection constructs**

The transfection constructs were obtained in four cloning steps:

1. Genomic PCRs of *sep2* and *sep3* 3’UTRs were cloned in a pGEX-6P-1 derivative, which contained the mcherry coding sequence (pGEX-cherry-backbone).
2. *Sep2* and *sep3* coding regions were inserted upstream the *mCherry*. Recombinant bacteria were analysed for the ability to produce the chimeric protein GST-SEP-CHERRY both by visual inspection with a fluorescence microscope and by SDS-PAGE of the IPTG induced bacterial colonies. Three promising clones for each transformation were sequenced.
3. The upstream regions of the two *sep* genes were cloned in the transfection vector A-B7red(F)SSU.
4. The *sep2- or sep3*-*mCherry*-3’UTR cassettes were excised from the plasmids obtained in cloning step 2 and inserted in the A-B7red(F)SSU vector downstream the upstream regulatory regions thus generating the final transfection constructs.

**Cloning step 1**

The 3’UTRs specific for *sep2* and *sep3* were amplified with the primers shown below, generating fragments of 743bp and 832bp respectively. PCRs were digested with SpeI-NotI and cloned in the same sites of the plasmid pGEX-cherry-backbone.

**Sep2 3’UTR**

3’-bis-spe: Gaccactagtgacgtctataattatccaaaccaataagacg

3’-bis-not: Gaccgcggccgcattgtagtactattattgcg

**Sep2 3’UTR**

3’-ter-spe: gaccactagtgacgtcGTAGATTCTGGTGGATATTATC

3’-ter-not: Gaccgcggccgccaatttaccataatcaaacag

**Cloning step 2**

Genomic PCRs of *sep2* and *sep3* coding regions (465 and 462 bp respectively) flanked by, EcoRI-HindIII and SmaI restriction sites were digested with EcoRI-SmaI and cloned in the same sites of the two plasmids obtained in the previous cloning step. The primers used are shown below. Recombinant bacterial colonies were grown in the presence of 1mM IPTG for three hours and then analysed for mCherry fluorescence; 80μl of bacterial suspension for each sample were analyzed in SDS-PAGE. Non-induced controls were run in parallel. Two colonies for each gene displaying fluorescent bacteria and expressing a recombinant protein of the expected size were chosen for sequencing.

**PCR *sep2* coding:**

sep1&2Hind-cod-for: gaccgaattcaagcttATGAAACTAGCAAAAGCATT

sepbisSma-cod-rev: gacccccgggattcaattttacagtatatgg

**PCR sep3 coding**

SepterHind-cod-for: gaccgaattcaagcttATGAAATTAGCAAAAGCATT

septerSma-cod-rev: gacccccgggcaaataacgtaattgatagcg

**Cloning step 3**

Two genomic PCRs, corresponding to *sep2* and *sep3*, upstream regulatory regions (1188 and 1253bp respectively) were amplified using the primers shown below. PCR fragments were digested with XhoI and HindIII and cloned in the same sites of the A-B/red(F)SSU transfection vector. The recipient plasmid contains a selection box based on the human *dhfr* under the control of the promoter of the elongation factor1 and the 3’UTR of the *P. berghei* dhfr. The *P. berghei* d-ssu-rrna as a target region for integration.

**Sep2 upstream regulatory region**

Bis-prom-sin: GaccctcgagGTGTATGAATTTAAGAATTTC

1&bisprom-Hind-rev: Gaccaagcttttttgtataagtaaaaaaattataat

**Sep3 upstream regulatory region**

1&ter-prom-sin: GaccctcgagCTAGTAATGGCAATAAATTGC

Ter-prom-Hind-rev: Gaccaagctttttcgtataattaagtaaaaaaa

**Cloning step 4**

The sep2-cherry-3’UTR (1936bp) and sep3-cherry-3’UTR (2022bp) cassettes from the cloning step 2 were excised by HindIII-NotI restriction and cloned in the same sites of the transfection vector containing the upstream regulatory regions obtained in cloning step 3. These final constructs were used in transfection experiments.
